# Molecular Dissection of *Cucumis metuliferus* Resistance against *Papaya Ringspot Virus* by Grafting

**DOI:** 10.3390/plants9121666

**Published:** 2020-11-27

**Authors:** Jen-Ren Chen, Shang-Ling Ou, Ting-Iun Nieh, Chih-Yu Lu, Hsin-Mei Ku

**Affiliations:** 1 Section of Biotechnology, Taiwan Seed Improvement and Propagation Station, Taichung City 426, Taiwan; jrchen0112@gmail.com; 2 Department of Agronomy, National Chung Hsing University, Taichung City 402, Taiwan; slou@dragon.nchu.edu.tw (S.-L.O.); nr556638@yahoo.com.tw (T.-I.N.); fishg1216@hotmail.com.tw (C.-Y.L.); 3 Advanced Plant Biotechnology Center, National Chung Hsing University, Taichung City 402, Taiwan

**Keywords:** *Cucumis metuliferus*, virus resistance, immune, RNA silencing, grafting

## Abstract

Vegetable crops of the genus *Cucumis* are very popular worldwide and have great market value. However, their fruit quality and yield are hindered by viral diseases. *C. metuliferus* is considered a wild species with resistance to viral diseases that is lacking in cultivated crops of the *Cucumis* genus, such as melon. The *C. metuliferus* line L37 shows extreme resistance against *Papaya ringspot virus* (PRSV-HA), whereas line L35 is a susceptible line. In this study, reciprocal grafting experiments between L35 and L37 were performed, and the PRSV-HA strain was pre-inoculated in the rootstock leaves. The results revealed that the resistance signal in the L37 rootstock could transmit and provide resistance to the L35 scion. Subsequently, double sandwich grafting was performed using the pre-inoculated L35 as the rootstock, which was then grafted onto the L37 intermediate and the L35 scion. The results showed that PRSV-HA RNA accumulated in the L35 rootstock leaf, petiole, and stem tissues, whereas PRSV-HA RNA accumulated in some intermediate and scion petiole and stem tissues. No HCPro RNA was detected in the L35 scion leaves. The results showed that the suppression of the virus occurred in the leaves, and the resistance effect spread from the rootstock in the scion direction. Hence, this study has demonstrated that RNA silencing of systemic signals is responsible for L37 resistance against PRSV. *C. metuliferus* L37 could provide a valuable resistance source for crops of the *Cucumis* species against viral diseases through grafting.

## 1. Introduction

*Cucumis metuliferus* (2n = 24) is a member of the *Cucumis* genus of the Cucurbitaceae family. *C. metuliferus* was grouped into the *Melo* subgenus of the Cucurbitaceae family based on the sequence similarity of six chloroplast loci and the nuclear ribosomal ITS1-5.8S-ITS2 regions [1,2]. The fruit of *C. metuliferus* is covered with stout, conical, blunt-based, and sharp stalk spines. Hence, it has several common names such as African horned melon, Kiwano, spiny cucumber, or recently, the commonly named devil fruit. It is consumed as a vegetable and is commercially grown for export due to its orange-reddish color and long shelf life. Widely distributed throughout the tropical and subtropical sub-Saharan regions of Africa [2], *C. metuliferus* is a monoecious annual species with climbing or sprawling vines [3]. It has been reported that *C. metuliferus* could provide resistance against root-knot nematodes [4], gummy stem blight [5], Fusarium wilt [6], and viral diseases caused by *Watermelon mosaic virus* (WMV), *Papaya ring spot virus* (PRSV) [7], and *Cucurbit yellowing stunting disorder virus* (CYSDV) [8].

Plants employ multiple defense mechanisms, such as the R (resistance) gene-mediated response and RNA silencing against pathogen infection. One of the common types of R-gene mediated defense responses is the induction of rapid localized cell death, which is highly recognizable by its necrotic lesions, called the hypersensitive response (HR). HR could be triggered by plant resistance proteins (R proteins), which recognize directly or indirectly virulence effectors from the virus, thereby activating the plant’s defense response. For example, the tobacco *N* gene encodes a nucleotide-binding site-leucine-rich repeat (NBS-LRR)-type protein that provides resistance against *Tobacco mosaic virus* (TMV) [9]. Alternatively, extreme resistance in plant hosts has been described with no apparent HR at the cellular level. This type of resistance response is called immune, in which no disease symptom or virus accumulation is detected [10].

Both DNA and RNA viruses can trigger plant antiviral defense responses, and RNA is the target of RNA silencing, also called posttranscriptional gene silencing (PTGS) [11]. In plants, dsRNA is synthesized as a virus replication intermediate structure that could in turn trigger RNA silencing. These virus-derived dsRNAs are first recognized and processed by RNase III-like enzymes Dicer-like (DCL)-4 and DCL-2, which cut the dsRNAs into short interfering RNA (siRNA) duplexes 21 and 22 nucleotides in length, respectively. After stabilization via HUA enhancer 1 (HEN1)-mediated methylation of their 3′ ends, these virus siRNAs are transported to the cytoplasm, where they are recruited by Argonaute (AGO) proteins to form a functional RNA-induced silencing complex (RISC) that acts to inactivate viral genomes [12,13,14].

Grafting is a traditional technique for asexual propagation and cultivation in tree and vegetable crops. It is accomplished most commonly by connecting two plant segments, the upper part known as the scion and the lower part called the rootstock. Early fruit agronomists noticed that certain rootstocks found in seedling populations could provide favorable characteristics for the scion, such as scion vigor, an improvement of fruit quality, climatic adaptability, and resistance to pests and diseases. To date, grafting has also been important for studying the function of proteins, RNAs, and hormones that act over long distances in plants. Transmissible signals from rootstock to scion or the opposite direction through phloem have been convincingly demonstrated [15,16]. For example, the long-distance transport of systemic RNA silencing signals was demonstrated by grafting experiments in transgenic tobacco plants [17].

PRSV-HA, a PRSV isolate from Hawaii, causes disease symptoms in the *C. metuliferus* line Acc. 2459 (hereafter called L35), including leaf chlorosis, a reduction of leaf size, and short internodes observed 7–10 dpi (days after inoculation) [7]. Another line, PI292190 (hereafter called L37), was reported to show multiple resistances against several viruses and pathogens [6,7,18]. Importantly, L37 showed an “extreme resistance” (or immune) type of defense response against PRSV-HA. No disease symptoms or viruses were detected in L37 plants. However, little is known about the mechanism of the extreme resistance of L37 against PRSV-HA. In this study, reciprocal grafting experiments between the L35 and L37 lines were conducted to dissect the resistance mechanism in L37. In addition, double sandwich grafting was performed with PRSV-HA pre-inoculated on the leaves of the rootstock. Disease symptoms and PRSV-HA RNA accumulation on the scion leaves were evaluated. This study is the first to show that the resistance responses of L37 rootstock against PRSV infection could provide systemic signals transmitting systemically to provide resistance in L35 scions.

## 2. Results

### 2.1. Reciprocal Grafting Between Susceptible L35 and Resistant L37

**Figure 1:**
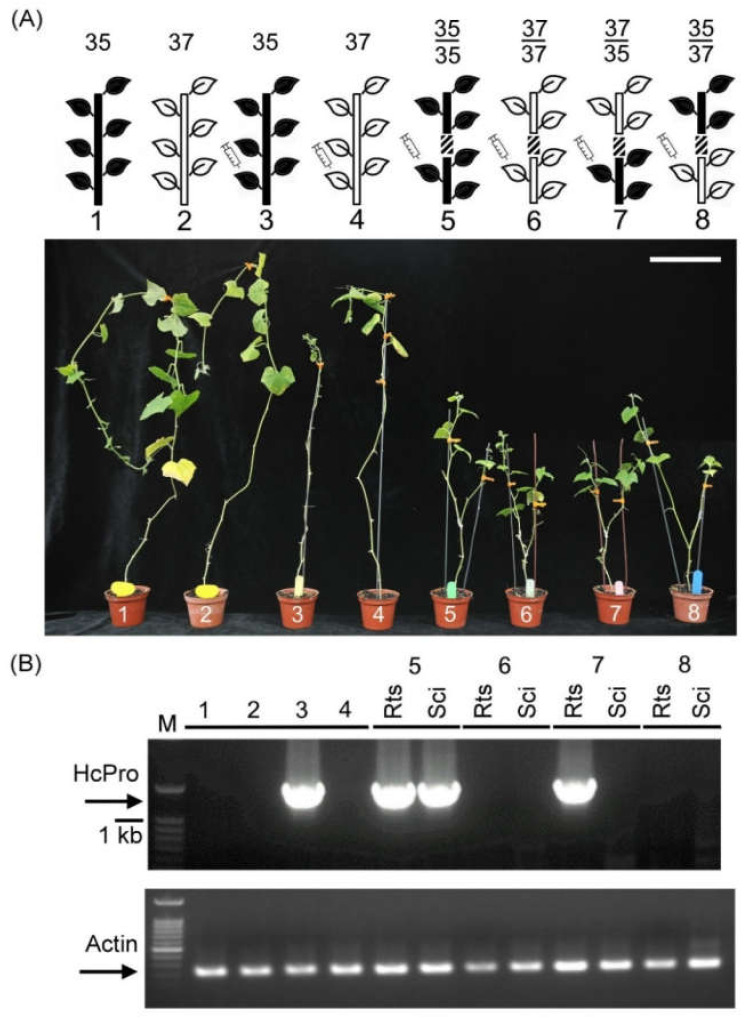
Nongrafting and reciprocal grafting of *C. metuliferus* against PRSV inoculation. (**A**) Schematic representation of reciprocal grafting between resistant *C. metuliferus* line PI292190 (L37) and susceptible line Acc.2459 (L35). 1–4 are nongrafted controls. 5–6 and 7–8 were self-grafting and reciprocal grafting, respectively. The PRSV-HA strain was inoculated at the rootstock, and the scion was grafted onto the rootstock. As a control, 1–2 were nongrafted, and no PRSV inoculation was performed. White plants indicate L35, and black plants indicate L37. The syringe indicates the PRSV-HA inoculation of the rootstock and the slash plug represents the grafted site. The white bar indicates 30 cm. (**B**) Analysis of PRSV HCPro RNA accumulation in the systemic leaves of the rootstock (Rts) and scion (Sci) grafting plants. Actin indicates the internal control; M indicates the molecular marker. n = 3.

To validate PRSV resistance and the influence of grafting on PRSV-HA transmission in L37 and L35 plants, self- and reciprocal-grafting experiments were performed. In nongrafting controls (L35 and L37), PRSV-HA was artificially inoculated on one-month-old seedlings (with two cotyledon leaves and the first true leaf). The PRSV-HA-infected L35 showed severe mosaic, malformation, and narrow leaf blades at 21 days post inoculation (dpi), whereas no symptoms were observed in the PRSV-HA-inoculated resistant control L37 (Figure 1A no. 3, 4). This verified that L35 was susceptible and that L37 showed extreme resistance against PRSV-HA infection. In the self-grafting experiments, one-month-old seedlings as rootstock were pre-inoculated with PRSV-HA, the shoot was deheaded at 10 dpi, and the scion was grafted onto the rootstock. The self-grafted L35 showed disease symptoms, but the self-grafted L37 did not (Figure 1A no. 5, 6), which was consistent with the nongrafted controls (Figure 1A no. 3, 4). These results indicated that grafting did not affect PRSV-HA transmission in the susceptible line L35 and the extreme resistance in line L37, as expected. Furthermore, reciprocal grafting experiments were conducted in which L37 as the scion was grafted onto L35 rootstock (Figure 1A no. 7) and, alternatively, L35 as the scion was grafted on L37 rootstock (Figure 1A no. 8). All of the rootstocks were pre-inoculated with PRSV-HA before grafting. The results showed that disease symptoms only appeared on the pre-inoculated L35 rootstock leaves, and no symptoms were observed on the L37 scion leaves (Figure 1A no. 7). However, no symptoms were observed on the rootstock and scion leaves when L37 was pre-inoculated as rootstock (Figure 1A no. 8). The results indicated that L37 could hinder the systemic transmission of PRSV-HA.

To monitor the existence of PRSV-HA, reverse transcription PCR (RT-PCR) of PRSV-HA’s HCPro gene was performed on the systemic leaves of the rootstock and scion. As shown in Figure 1B no. 3 and 5, PRSV-HA was only present in the systemic leaves of the self- and nongrafting controls of L35 inoculated with PRSV-HA. While L35 was pre-inoculated as rootstock, PRSV-HA was detected in the rootstock leaves but not in the L37 scion (Figure 1B no. 7). This confirmed that grafting did not restrict PRSV systemic infection in L35. On the other hand, no PRSV-HA was detected in the L35 and L37 scions grafted onto the pre-inoculated L37 rootstock. This confirmed that L37 could suppress PRSV-HA systemic spread (Figure 1B no. 6, 8). These results showed that L37-resistant or L35-susceptible phenotypes in nongrafting or grating experiments were consistent with the RT-PCR results. Moreover, the resistance of the L37 scion against PRSV-HA indicated that the resistance of L37 did not require the presence of root tissues (Figure 1B no. 7).

### 2.2. PRSV-Induced L37 Systemic Resistance in Grafting

**Figure 2:**
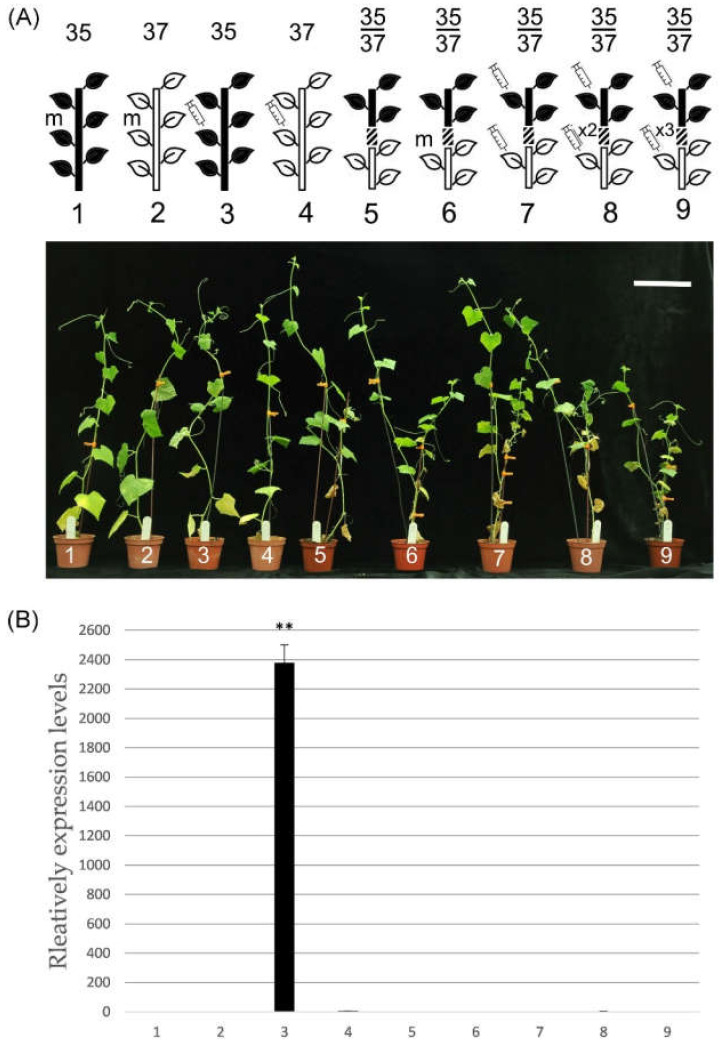
PRSV induced systemic resistance in L37 rootstock. (**A**) Schematic and grafted plants after PRSV-HA inoculation. Nos. 1 and 2 are L35 and L37 nongrafted plants inoculated with the mock control. Nos. 3 and 4 are L35 and L37 nongrafted plants inoculated with PRSV-HA. Nos. 5–9 are L35 scions grafted onto L37 rootstocks. No. 5 is a grafted plant with a noninoculated control, and No. 6 is a grafted plant inoculated with a mock control. No. 7–9 are L35 scions grafted onto L37 rootstock and then inoculated into L37 rootstock one to three times by PRSV, and the L35 scions were inoculated the same time as the last L37 rootstock inoculation. Schematic drawing and symbols are as in Figure 1. ×2 and ×3 indicate PRSV-HA inoculated in the L37 rootstock twice and three times at two-day intervals, respectively. The white bar indicates 30 cm. (**B**) qRT-PCR analysis of PRSV-HA HCPro RNA accumulation in the scion L35 systemic leaves. Total RNA was extracted from the systemic leaves of four-week-old nongrafted or grafted *C. metuliferus* plants 7 dpi. Relative amount of HCPro RNA was normalized to Actin mRNA. Values represent the fold change, relative to the negative control, as means ± standard deviation from three biologically independent experiments. Asterisks indicate statistically significant difference (** *p* < 0.01 by Student’s *t*-test).

To test whether the systemic resistance signal in L37 induced by PRSV-HA could transmit and provide resistance to the scion, the L35 scion was grafted onto one-month-old seedling L37 rootstock, in which the rootstock was inoculated with PRSV-HA one to three times within two-day intervals. Most importantly, the L35 scion was also inoculated at the last inoculation in all grafted plants. In the nongrafted controls, plants inoculated by mock (Figure 2A no. 1, 2) as a negative control and PRSV-HA as positive controls (Figure 2A no. 3, 4) were performed. In the grafted controls, noninoculation and mock inoculation were used as negative controls (Figure 2A no. 5, 6). The disease symptom was recorded and the expression of PRSV *HCPro* gene in the L35 scion leaves at 7 dpi was evaluated by quantitative Real-Time PCR (qRT-PCR) (Figure 2A,B no. 7–9). The results showed that the disease symptoms and the HCPro RNA accumulation were only observed in the nongrafted L35 positive controls (Figure 2A,B no. 3), whereas no symptoms or HCPro RNA accumulation were detected in any of the other tested L35 scions (Figure 2A,B no. 5–9). Moreover, no apparent difference was observed in the L35 scions among the grafted plants inoculated with PRSV-HA one, two, and three times on the L37 rootstocks (Figure 2B no. 7–9). Comparing the grafted experiments of Figure 1 and Figure 2, the L35 scions were inoculated by PRSV-HA in Figure 2 no. 7–9, but not in Figure 1 no. 8. No symptoms or virus RNA were detected in the plants of Figure 1 no. 8, which indicated that pre-inoculated PRSV-HA in the L37 rootstock would not be able to be transmitted to the L35 scion. However, this could still not provide evidence of any resistance signal transmitted from the L37 rootstock to the L35 scion. On the other hand, as shown in Figure 2 no. 7–9, the results showed that the resistance signals from the L37 rootstock were able to transmit and provide resistance in the L35 scion, since there was PRSV-HA inoculation in the L35 scion. Furthermore, this also revealed that once generated in the L37 rootstock induced by the first PRSV-HA inoculation, the resistance signals could transmit over long distances and successfully provide systemic resistance to the L35 scion.

### 2.3. Dissection of Systemic Resistance Signal Transmission by Double Sandwich Grafting

**Figure 3:**
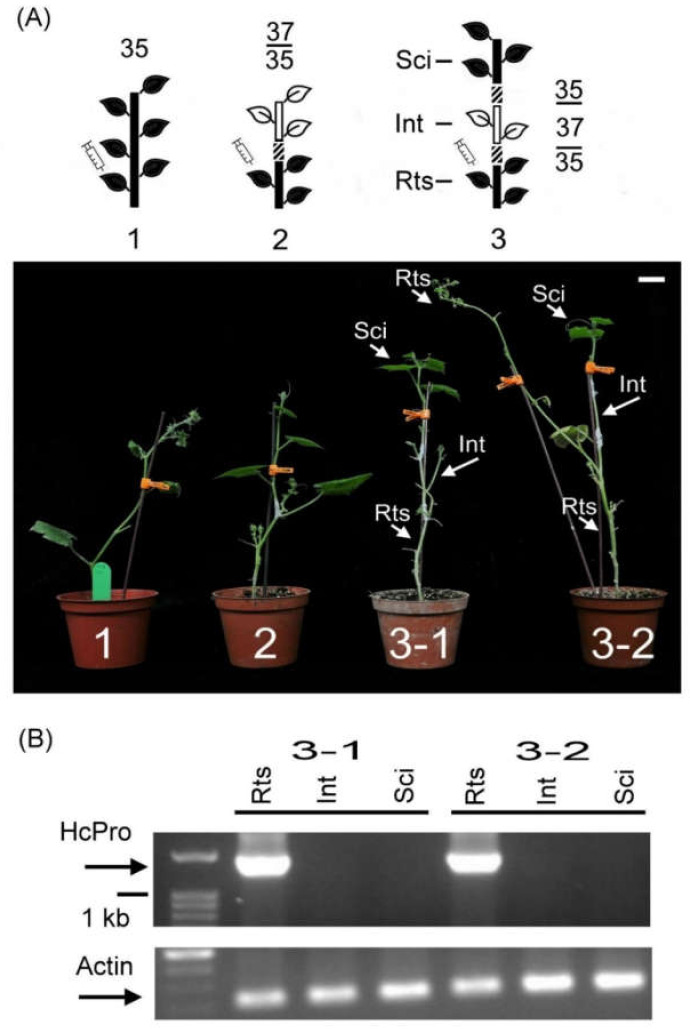
Graft transmission of virus suppression in the scions. (**A**) Schematic and double sandwich grafted on PRSV-HA pre-inoculated L35 rootstock. 1 is the nongrafted L35 inoculated with PRSV-HA. 2 is the L37 scion grafted onto the pre-inoculated L35 rootstock. 3 is a double sandwich grafting plant. The intermediate L37 and scion L35 were grafted onto the pre-inoculated L35 rootstock in turn. The schematic drawing and symbols are as represented in Figure 1. Rts, Int, and Sci indicate the rootstock, intermediate, and scion, respectively. The white bar indicates 10 cm. (**B**) Analysis of PRSV HCPro RNA accumulation in the double sandwich grafting plants. Actin indicates the internal control; M indicates the molecular marker.

To further study the effect of the systemic resistance signals derived from the L37 rootstock through grafting in detail, double sandwich grafting experiments were performed. The pre-inoculated PRSV-HA L35 was used as the rootstock. The L37 intermediate and the L35 scion were grafted onto the PRSV-HA pre-inoculated L35 rootstock (Figure 3A no. 3). The nongrafted L35 (Figure 3A no. 1) and L37 grafted onto the pre-inoculated L35 (Figure 3A no. 2) were used as controls. In double sandwich grafting, no symptoms were observed in the L35 scion and L37 intermediate 10 days after grafting, whereas disease symptoms were observed in the pre-inoculated L35 rootstock (Figure 3A no. 3-1 and 3-2). Meanwhile, PRSV-HA RNAs were detected in the L35 rootstock but not in the L37 intermediate or the L35 scion (Figure 3B). In addition, no recovery phenomenon was observed in the L35 rootstock. In summary, the results shown in Figure 3 indicate that the L37 intermediate could efficiently hinder PRSV-HA transmission into the upper leaves of the L35 scion.

**Figure 4:**
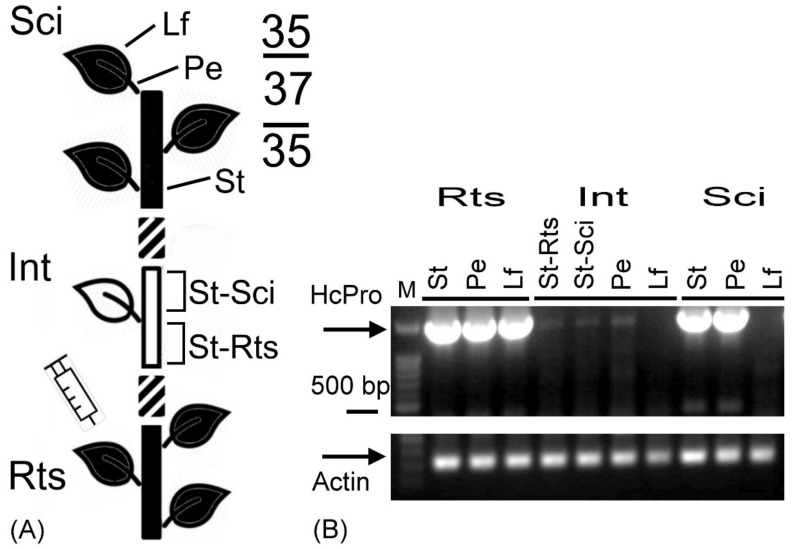
Dissection of systemic PRSV suppression transmitted in the vascular system of double sandwich grafting plants. (**A**) Schematic of double sandwich grafting. The intermediate L37 and scion L35 were grafted onto the pre-inoculated L35 rootstock in turn. Each plant part was subdivided into leaf (Lf), petiole (Pe), and stem (St) tissues. The schematic drawing and symbols are represented as in Figure 1. (**B**) Analysis of PRSV HCPro RNA accumulation in double sandwich grafting plants. Actin indicates the internal control; M indicates the molecular marker.

Furthermore, the PRSV-HA HCPro RNA in the stem, petiole, and leaf tissues of the plants in the double sandwich grafting was surveyed. The results showed that HCPro RNA was detected in the leaf, petiole, and stem tissues of the pre-inoculated L35 rootstock as expected (St, Pe, Lf of Rts in Figure 4B). HCPro RNA was also detected in the stem and petiole of the L35 scion (St, Pe of Sci in Figure 4B), and very trace amounts of virus were detected in the L37 intermediate (St-Rts, St-Sci, and Pe of Int in Figure 4B). The results clearly indicated that L37 did not restrict the systemic movement of PRSV-HA in the vascular tissues, which could also arrive at the stem and petiole of the L35 scion. Most importantly, PRSV-HA was not detected in the leaf tissues of the L37 intermediates and L35 scions (Lf of Int and Sci in Figure 4B) and consequently indicated that the systemic resistance signal derived from the leaf of the L37 intermediate could be transmitted to the leaf of the L35 scion in which PRSV-HA was silenced, and no symptoms were observed.

**Figure 5:**
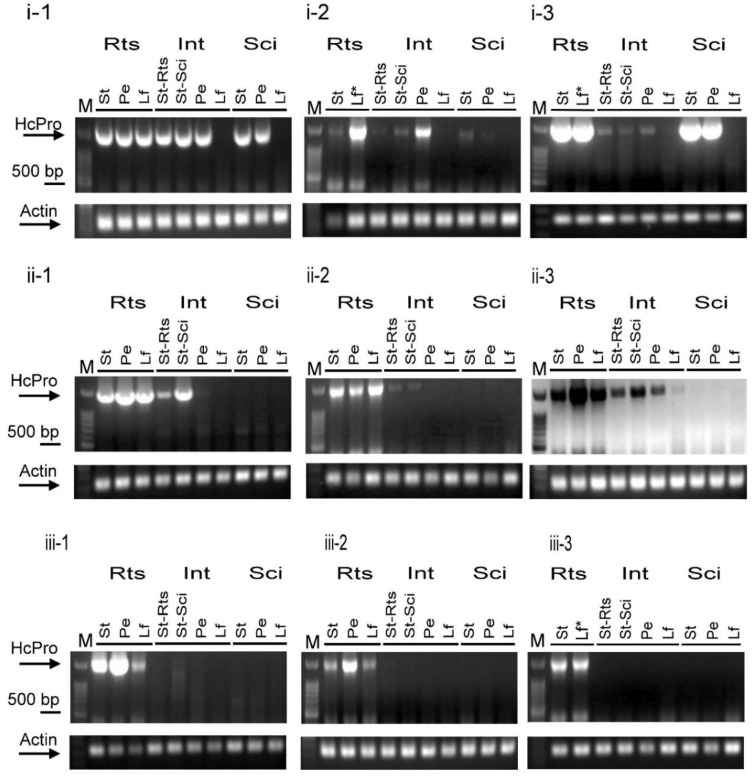
Three PRSV RNA accumulation patterns were revealed in the double sandwich grafting plants. The intermediate (Int) L37 and scion (Sci) L35 were grafted onto pre-inoculated L35 rootstock (Rts) in turn. Each plant part was subdivided into leaf (Lf), petiole (Pe), and stem (St) tissues. Analysis of PRSV HCPro RNA accumulation in double sandwich grafting plants revealed three patterns in which three plants are shown (-1, -2, -3): (**i**) PRSV accumulated in the L35 rootstock and in petiole and stem tissues of intermediate L37 and scion L35. (**ii**) PRSV accumulated in the pre-inoculated L35 rootstock and in the petiole and stem tissues of the intermediate L37. In the ii-3 grafting plants, trace amounts of the virus were detected in the leaves of the L37 intermediate. The gel image was strengthened by inverting it. (**iii**) PRSV accumulated only in the pre-inoculated L35 rootstocks, and no virus was detected in the intermediate L37 and scion L35. Each gel photo represents a different biological replicate. Lf* indicates severe malformation of the leaf and petiole were combined into one sample. Actin indicates the internal control; M indicates the molecular marker.

In Figure 4, fainter bands of PRSV-HA HCPro RNAs were observed in the stem and petiole tissues of intermediate L37 than in those of the L35 rootstock or the L35 scion. To exclude the possibility of a technical error, more biological replicates were tested. As shown in Figure 5, three different patterns of PRSV-HA HCPro RNAs were observed among the double sandwich grafting plants. (i) HCPro RNAs were detected in all pre-inoculated L35 rootstock tissues and in the petiole and stem tissues of the L37 intermediate and the L35 scion. No PRSV was detected in the leaf tissues of the L37 intermediate and L35 scion (Figure 5(i-1–i-3)), and this was the same as the result shown in Figure 4. (ii) HCPro RNAs were detected in all pre-inoculated L35 rootstock tissues and in the petiole and stem tissues of the L37 intermediate. No PRSV was detected in the leaf tissues of the L37 intermediate or in all of the tissues, including the petiole, stem, and leaf tissues of the L35 scion (Figure 5(ii-1–ii-3)). (iii) HCPro RNAs accumulated only in the pre-inoculated L35 rootstocks. No PRSV was detected in any tissues of the L37 intermediate or the L35 scion (Figure 5(iii-1–iii-3)). Moreover, the amount of HCPro RNA in the petiole and stem tissues of the L37 intermediate was much less abundant than that in the L35 rootstock in five out of 10 double sandwich grafting plants (Figure 4 and Figure 5(i-2, i-3, ii-2, ii-3)). Overall, no PRSV-HA was detected in leaf tissues of either the L37 intermediate or the L35 scion, which was highly consistent, as shown in Figure 5. However, with only one exception, trace amounts of HCPro RNAs were detected in leaf tissues of the L37 intermediate, as shown in Figure 5(ii-3), which was especially enhanced by inverting the image. These results indicated that the intensity of the induced systemic resistance in L37 was not completely identical among the double sandwich grafting plants. Most importantly, the induced L37 resistance signals could transmit and achieve 100% systemic silencing of PRSV-HA RNA in the leaves of the L35 scions.

In summary, these results provided evidence that the initiation and transmission of resistance signals against PRSV-HA did not require root tissue (Figure 1 and Figure 4), systemic resistance could be induced by a single PRSV-HA inoculation (Figure 2), the direction of the systemic resistance signals were transmitted from the rootstock to the scion (Figure 3), and viruses were suppressed (silenced) in the leaf tissues but not in the petiole and stem tissues of the L37 intermediate and L35 scion (Figure 4 and Figure 5). Hence, the RNA silencing occurred in L37, in which the resistance signal could transmit and provide a very high level of systemic extreme resistance against PRSV-HA infection in the L35 scion through grafting.

## 3. Discussion

In contrast to other plant pathogens, viruses are obligate parasitic organisms, and they enter host cells through mechanical inoculation or infection with the aid of vectors such as insects to overcome the cell wall barrier of plant hosts. After entry into plant cells, viruses face two major defense mechanisms, namely, the plant single dominant R gene-mediated response and the RNA silencing machinery, which may cooperate with each other in the early stages of the plant hosts against virus infection (as reviewed in [19]). First, studies of the R gene-mediating defense response have been well documented in recent decades. HR is commonly observed in conjunction with the R protein-mediated defense response. However, extreme resistance, such as L37 resistance, in this study, where no HR occurred and no symptoms were detected in the resistant hosts has been documented, but in a much smaller number of reports. For example, the tomato dominant R gene *Ty-1*, encoding RdRp, was reported to provide extreme resistance in tomato against TYLCV. Other examples include tomato *Tm-1* against ToMV (*Tomato mosaic virus*) and RTM proteins against potyviruses in the host *A. thaliana* [19].

In this study, no symptoms or viral RNA were observed in the nongrafted control *C. metuliferus* resistance line L37 (Figure 1 no. 4 and 6) and the grafted L37 scion where L35 was used as the rootstock and was inoculated with PRSV-HA (Figure 1 no. 7). This further confirmed that extreme resistance did occur in L37. In a previous report, a single dominant gene, *Wmv*, in L37 against PRSV-HA has been proposed [7]. Furthermore, using cDNA-AFLP, the *serine proteinase inhibitor* (*SPI*) gene was identified as differentially expressed between susceptible L35 and L37 and could potentially be one of the candidates of *Wmv* [20]. Subsequently, in transgenic L37 plants carrying the gene silencing construct of the *serine proteinase inhibitor* (*SPI*), the disease symptoms were observed at 21 dpi after PRSV-HA infection. This suggests that SPI might be one of the components required for PRSV-HA resistance in L37 [21]. However, no overexpression of the *SP1* gene in transgenic susceptible L35 has been reported, which supports the idea that the *SP1* gene fragment from L37 could provide resistance for L35. Therefore, the single dominant gene *Wmv* remains unidentified, and little is known about the extreme resistance mechanism in L37.

Second, RNA silencing is another well-documented resistance mechanism in plant hosts against virus infection. It is well known that RNA silencing is a fast and efficient method of anti-viral resistance, including the suppression of virus replication and short- and long-distance movement. Several grafting studies have shown that RNA silencing signals could be transmitted and provide antiviral resistance from rootstock to scion in transgenic or nontransgenic plants [22,23,24]. This study also employed grafting experiments to test whether any resistant signal derived from L37 rootstock could transmit successfully and provide resistance in the L35 scion against PRSV-HA. In (Figure 1 no. 8), no symptoms or virus RNA was detected in the L35 scion and rootstock when PRSV-HA was inoculated in the L37 rootstock. This result indicated that the PRSV-HA virus was suppressed in L37 rootstock. Furthermore, when the L35 scion was grafted onto the L37 rootstock, PRSV-HA was pre-inoculated on the L37 rootstock one to three times, and the L35 scion was also inoculated at the last inoculation, no symptoms occurred in L35 scion (Figure 2). These results indicated that there must be resistance signals in L37 that could be transmitted and provide resistance to the L35 scion, even after it was inoculated with PRSV-HA (Figure 2B no. 7–9). These results indicate that RNA silencing might also play an important role in L37 resistance against PRSV-HA, similar to the previous grafting studies mentioned above.

In addition, additional evidence of RNA silencing responsible for the L37 resistance mechanism is evident in studies of the PRSV-HA 5-1 strain. Interestingly, PRSV-HA 5-1, a nitrous acid-induced attenuated mutant of PRSV-HA, was shown to break down the extreme resistance, and it showed symptoms in L37, but it was reported to be symptomless or only cause a mild infection in L35 [25]. The HCPro protein of the genus *Potyvirus* is a well-known viral proteinase essential for *Potyvirus* infection. Moreover, the HCPro protein also suppresses RNA silencing via directly binding to siRNAs and prevents virus RNA silencing by inhibiting RISC assembly [26]. Since the PRSV-HA 5-1 strain with the mutated *HCPro* gene could overcome the immunity of L37, this further implies that RNA silencing would be the important mechanism responsible for L37 resistance against PRSV-HA.

Furthermore, disease symptoms and PRSV-HA RNA were detected, and no recovery was consistently found in the L35 rootstock in the double sandwich grafted plants (Figure 3, Figure 4 and Figure 5). This indicated that the RNA silencing signal was spread one-way only from the rootstock to the scion in the double sandwich grafted plants. This is different from previous studies showing that a bidirectional movement of the silencing signal was found in transgenic grafted tobacco plants [27,28]. The difference might be caused by transgenic plants with strong promoters, such as the 35S promoter, being applied in grafting in these studies, whereas in this study, the nontransgenic resistance line L37 with native regulatory units was used in grafting. Furthermore, another report proposed that silencing signals spread systemically via the phloem and followed the source-sink direction [29]. Hence, it is more likely that a one-way direction of RNA silencing signals transported from the lower leaves of the L37 intermediate as a source to the upper part of the young shoots (the top of the L35 scion) acted as a sink in grafted plants in this study. Alternatively, the leaves of the L35 rootstock might be too old, or the symptoms might be too severe, and insufficient numbers of young leaves could be evaluated; consequently, no recovery was found in the L35 rootstock.

PRSV-HA RNA accumulation in double sandwich grafted plants showed different types of patterns in this study (Figure 4 and Figure 5). In general, this study showed that antiviral RNA silencing was more obvious in leaves of the L37 intermediate and L35 scion than in other tissues in the double sandwich grafted plants. In a previous study, nonsilenced scions were grafted onto silenced rootstocks of tobacco plants, and some scions could be infected by PVX, indicating that grafting transmission silencing was not 100% efficient [30]. Moreover, double silencing *NtTOM1*/*NtTOM3* transgenic tobacco (Sd1 line) showed resistance to *Tomato mosaic virus* (ToMV) and *Tobacco mosaic virus* (TMV) by silencing the genes of two host factors, *NtTOM1* and *NtTOM3*, supporting tobamovirus multiplication. The nonsilenced tomato and tobacco scions grafted onto the Sd1 line showed 50% and 60% degrees of silencing in the scions, and siRNAs were detected in the Sd1 rootstock and the grafted scions. This result also indicated that systemic RNA silencing is not 100% efficient and that the presence of the siRNAs was consistent with silencing in the scions [22]. In summary, different grafting combinations or grafted individuals would show variations in systemic RNA silencing.

In plants, viral dsRNA in the host triggers RNA silencing as a defense mechanism against viruses. These dsRNAs are rapidly processed into small RNAs (sRNAs) of 21 to 28 nucleotides (nt) in length, which then interact with RISC and ultimately give rise to secondary sRNAs for target gene RNA silencing. RNA silencing generates systemic signals that spread from the initiation cell to neighboring cells through plasmodesmata and systemically spread over long distances through the phloem tissue [12,31]. Hence, sRNA sequencing was performed in inoculated leaves of susceptible L35 and resistant L37 inoculated with PRSV-HA at 2 dpi. The preliminary results revealed that some sRNAs were differentially accumulated between L35 and L37. However, whether these sRNAs are directly involved in RNA silencing or virus resistance still needs more experiments for validation (data not shown).

In double sandwich grafted plants, pre-inoculated L35 was used as a rootstock to constantly provide PRSV-HA stimulus to L37. The results clearly showed that the L37 resistance signal did not restrict the systemic movement of PRSV-HA, which could be found in the stem and petiole tissues, but PRSV-HA was suppressed in the leaves of the L37 intermediate and the L35 scion (Figure 4 and Figure 5). These results indicated that RNA silencing might confer PRSV resistance in L37 and that systemic resistance signals were transmitted to susceptible L35 scions with very high efficiency. This phenomenon was first revealed in crops in the genus *Cucumis* and could support virus-resistant breeding programs for cultivated melon or other *Cucumis* crops.

Wild relatives are commonly considered to be valuable resources for the genetic improvement of cultivated varieties. *C. metuliferus* is considered to be the most primitive species among the cultivated *Cucumis* species based on its external fruit characteristics and flesh bitterness taste [32]. However, *C. metuliferus* has not been commonly used in cultivated melon improvement to date due to the difficulty of sexual hybridization between *C. melo* and *C. metuliferus* [33]. Alternatively, *C. metuliferus* were reported to be grafting compatible with cultivated melons [18,34]. *C. metuliferus* has been used as a rootstock for grafting to cultivated melons to improve root-knot nematode resistance. After being grafted onto *C. metuliferus* rootstocks, the susceptible melon lines showed the lowest root galling and nematode egg densities compared with the nongrafted lines. In addition, grafting showed no significant impact on the fruit quality of the tested lines. Hence, we propose that grafting susceptible scions onto L37 rootstock might help prevent virus infection without the need for transgenic plants; hence, *C. metuliferus* could be a valuable resistance resource for breeding resistant cucurbit crops.

## 4. Materials and Methods

### 4.1. Plant Materials and Virus Inoculation

*C. metuliferus* susceptible line Acc. 2459 (L35) and resistant line PI292190 (L37) seeds were germinated in pots and grown in a temperature-controlled greenhouse at 25 °C for one month (third true-leaf stage). Viruses were inoculated on two cotyledons and the first leaf with a PRSV-HA strain. The virus inoculum source was kindly provided by Dr. Fuh-Jyh Jan (National Chung Hsing University). The plants were mechanically inoculated with fresh inoculum sap diluted 1:50 (*w*/*v*) in sodium phosphate buffer (0.01 M, pH 7.0). Inoculated plants were kept in the same greenhouse for subsequent symptom observations and virus detection.

### 4.2. RT-PCR and qRT-PCR

The presence of PRSV and its quantity were monitored using the expression level of *HC-Pro* gene expression by RT-PCR and qRT-PCR analysis. The extraction of total RNA from 100 mg leaf tissues was carried out using TriPure isolation reagent (Roche Applied Science, Mannheim, Germany) according to the manufacturer’s instructions. The RNA quantity and quality were determined by using spectrophotometry and gel electrophoresis. One microgram of total RNA was used for the first-strand cDNA synthesis reaction consisting of 1 × M-MLV buffer, 1 unit of M-MLV reverse transcriptase (Protech Technology Enterprise, Taipei, Taiwan), 0.2 mM dNTPs, 0.1 mM primers, and 2.5 unit RNaseOUT (Invitrogen, Carlsbad, CA, USA). The reaction was performed in a thermal cycler under the following conditions: 60 min at 42 °C and then 10 min at 72 °C. PRSV HC-Pro-specific primers (forward: 5-AGAATGACGTGGCTGAAAAATTC-3; reverse: 5-CGCCGACAATGTAGTGCTTCAT-3) and *beta*-*actin* gene primers of *C. metuliferus* (forward: 5-ATCCACGAAACTACTTACAACTCC-3, reverse: 5-ATAGACCCTCCAATCCAGACAC-3) were adapted from previous studies [20,35]. The *beta*-*actin* gene was used as an internal control in each RT-PCR or qRT-PCR analysis. For RT-PCR, it consisted of 20 μL total volume PCRs, consisting of 1 × PCR buffer, 0.2 unit of ProTaq polymerase (Protech Technology Enterprise, Taipei, Taiwan), 0.2 mM dNTPs, 0.15 mM primers, and 1 µL first strand cDNA templates. All amplifications were performed in a thermal cycler under the following conditions: 2 min at 94 °C, followed by 30 s at 94 °C, 30 s at 55 °C for annealing temperature, 1 min at 72 °C for 35 cycles, and 5 min at 72 °C for a final extension. The PCR products were electrophoresed and visualized on 1% agarose gels. For qRT-PCR, 10 μL total volume PCRs consisting of 1 × PCR buffer, 0.025 unit of WizPure™ Taq polymerase (Wizbiosolution, Seongnam, Korea), 0.05 mM dNTPs, 0.6 μM primers, and 3 µL 1/5X first strand cDNA templates. The sequences of the qRT-PCR primers were as follows: PRSV HC-Pro-specific primers (forward: 5-TGAATGCGCGGAACATGAACGA-3; reverse: 5-GCGAACCTTTGATGAGCGTGTTG-3, designed from Primer3 v. 0.4.0 (https://bioinfo.ut.ee/primer3-0.4.0/) and *beta*-*actin* gene primers of *C. metuliferus* (forward: 5-ATCCACGAAACTACTTACAACTCC-3, reverse: 5-ATAGACCCTCCAATCCAGACAC-3) were adapted from previous studies [35]. All amplifications were performed in a CFX 96 Real-Time System (Bio-Rad, Hercules, CA, USA): 5 min at 94 °C, followed by 30 s at 94 °C, 30 s at 60 °C for annealing temperature, and 30 s at 72 °C for 50 cycles. Experiments were repeated three times independently. The results were recorded by the Bio-Rad CFX Manager Software version 3.0 (Bio-Rad, Hercules, CA, USA) and the data were averaged.

### 4.3. Plant Grafting

Two grafting and virus inoculation methods were used. (i) Plants that were approximately one month old were used for grafting. The clef grafting method was employed [17]. Rootstock was pre-inoculated by the PRSV-HA strain on cotyledons and leaves, and the terminal apex was removed at 7 dpi. Scions were prepared by removing their leaves and trimming the base of the scion to a V-shaped wedge. The scion/rootstock junction was secured with Parafilm and held together with a grafting clip. Grafted plants were covered with thin transparent plastic bags to avoid dehydration. The bag was removed 4 days after grafting. The presence of disease symptoms and virus was determined 10 days after grafting. (ii) Grafted plants of the L35 scion grafted onto L37 were used to manifest the presence of VIGS in L37. Virus was inoculated on the L37 rootstock one to three times in three-day intervals and then inoculated on the scion L35. The presence of disease symptoms and viruses in the systemic leaves of the L35 scions was determined at 7 dpi.

Double sandwich grafts were performed as the first grafting and virus inoculation method. Graft inoculation involved the grafting of healthy scions onto the infected rootstock. Scion L35 and the intermediate L37 were grafted on the pre-inoculated L35 rootstock in turn. The presence of disease symptoms and viruses in the leaf, petiole, and stem tissues were determined 10 days after grafting by using RT-PCR analysis.
